# A SIFT-Based DEM Extraction Approach Using GEOEYE-1 Satellite Stereo Pairs

**DOI:** 10.3390/s19051123

**Published:** 2019-03-05

**Authors:** Ioannis N. Daliakopoulos, Ioannis K. Tsanis

**Affiliations:** 1 Department of Environmental Engineering, Technical University of Crete, 73100 Chania, Greece; tsanis@hydromech.gr; 2 Department of Civil Engineering, McMaster University, Hamilton, ON L8S 4L8, Canada

**Keywords:** digital elevation model, algorithm, satellite stereo pair, SIFT, RANSAC

## Abstract

A module for Very High Resolution (VHR) satellite stereo-pair imagery processing and Digital Elevation Model (DEM) extraction is presented. A large file size of VHR satellite imagery is handled using the parallel processing of cascading image tiles. The Scale-Invariant Feature Transform (SIFT) algorithm detects potentially tentative feature matches, and the resulting feature pairs are filtered using a variable distance threshold RANdom SAmple Consensus (RANSAC) algorithm. Finally, point cloud ground coordinates for DEM generation are extracted from the homologous pairs. The criteria of average point spacing irregularity is introduced to assess the effective resolution of the produced DEMs. The module is tested with a 0.5 m × 0.5 m Geoeye-1 stereo pair over the island of Crete, Greece. Sensitivity analysis determines the optimum module parameterization. The resulting 1.5-m DEM has superior detail over reference DEMs, and results in a Root Mean Square Error (RMSE) of about 1 m compared to ground truth measurements.

## 1. Introduction

Land surface texture and topography regulate and interact with climatic, hydrological, geomorphological, and ecological mechanisms [1], and provide the foundation for socioeconomic processes to build upon. This coupling is so fundamental [2] that scientific observation can often be conceptualized solely by understanding the characteristics of the land surface upon which they are measured. Digital land surface, object models, and their statistical properties benefit from a wide range of state-of-the-art applications and environmental research topics, ranging from the estimation of terrestrial [3] and climatic variables [4] to hazard assessment [5] and disaster management [6], among others.

Remote sensing techniques have provided indispensable solutions for generating the Digital Elevation Models (DEMs) of various resolutions and accuracies [7], especially for large area coverage. Low-resolution DEM products (30 to 100 m) can also be adequate for numerous environmental applications [8] but provide poor terrain detail, especially in lowlands with minor slopes. GeoEye-1 currently has the highest commercial imaging system resolving power, and can collect samples at a ground resolution of 0.41 m in the panchromatic or black and white mode as well as multispectral or color imagery at a resolution of 1.65 m. The United States (US) government operation license regulation requires GeoEye’s products to be resampled at 0.5 m for all customers.

The intended use of the GeoEye GeoStereoTM product [9] is to obtain an accurate Digital Elevation Model (DEM) generation for three-dimensional (3D) viewing and feature extraction applications. Experiments on generating DEMs from GeoEye-1 were only made available recently [10]. Notable examples include comparisons with lower (e.g., [11] for various locations in Catalonia, Spain) and similar resolution satellite imagery (e.g., [12,13] for the coast of Almería, Spain and [14] for the city of Trento, Italy), as well as specialized environmental applications such as the production of 1:5000 topographic maps for Nanisivik, Nunavut, Canada [15], floodplain mapping of a seasonal stream in Crete, Greece [16], gully erosion assessment in Sehoul, Morocco [17], and enhancing the security of high-value facilities via earth observation data analysis at the Jülich Research Centre, Germany [18].

Due to misinterpretations caused by ambient light and shadow as well as sensor geometry, matching feature pixels in a stereo pair presents a challenge. Feature detection methods that initially detect pronounced features in images (e.g., corners, high entropy regions, scale space maxima, etc.), following local approaches [19,20,21] have demonstrated considerable success in a variety of applications such as object recognition [22], wide-base line stereo [23], robot navigation [24], content-based image retrieval [25,26], image stitching for panorama construction [27], etc. The Scale-Invariant Feature Transform (SIFT) proposed by [22,28] is probably the most popular and widely used local approach [29]. Besides SIFT, several methods, such as Principal Component Analysis (PCA)-SIFT [30], Gradient Location and Orientation Histogram (GLOH) [31], Speeded Up Robust Features (SUFR) [32], DAISY [33], Maximally Stable Extremal Regions (MSER) [23] and others, have been used for density matching in various applications. Previous evaluations and comparison [31,34] demonstrate the excellent performance of SIFT; nevertheless, a comparison to other approaches is beyond the scope of this research.

With new methods or modification to existing ones, researchers are still engaged with feature extraction and matching, especially for photogrammetric applications, with SIFT-like algorithms dominating the literature [35]. The SIFT algorithm or variants are successfully used in various DEM extraction applications, among which recent and captivating initiatives of topography extraction from Synthetic Aperture Radar (SAR) images [36], high accuracy lunar surface reconstruction from the Apollo 16 Mapping Camera (MC) [37], and archeological site topographic modeling from a compact camera onboard an Unmanned Aerial Vehicle (UAV) [38].

The exploration of the data sources’ efficiency in combination with a specific application and advances in available processing methods are still in demand [39] and offer new opportunities for research. The motivation for this work lies in the need to develop a new DEM extraction module that can allow the testing of feature extraction algorithms (such as SIFT presented in the paper) and fine-tuning their parameters beyond the black box methodologies provided by commercial software. Additional functionalities include DEM extraction from part of the stereo pair rather than the full dataset, as well as the ability to optimize and control processes in all parts of the methodology. Additionally, the module provides a flexible method of extracting area specific, high-resolution DEM products from stereo-pair imagery for specialized environmental applications that do not require a full-scale commercial software suite. The novelty and main contributions of this work include the use of parallel processing and image segmentation to decrease image processing time, and that besides previous work by the authors [40,41,42], to our knowledge, the combination of methods presented here for DEM generation from GeoEye-1 imagery has not been previously documented in the literature.

## 2. Methodology

### 2.1. Ground to Image

The first requirement for a DEM from satellite imagery is a satellite stereo-pair accompanied by a sensor model that describes the geometric relationship between the three-dimensional object space R(X,Y,Z) and two-dimentional image space M(r,c). The sensor model can be made available in either the rigorous physical sensor model or an abstract sensor model [43]. The proprietary physical sensor model includes all of the internal and external (i.e., location and orientation) sensor model information associated with a specific satellite sensor, at the moment the imagery is being captured but lacks Ground Control Point (GCP) information [44]. Instead of distributing a proprietary physical sensor model, vendors usually provide an approximation [45] via an abstract model or Rational Function Model (RFM) that also incorporates the use of vendor GCP information. The RFM is commonly defined by 78 rational function coefficients (RFCs), approximating the specific sensor model information to map geodetic ground points to the imaging system’s pixel coordinates. The advantage of the RFM is that it is sensor-independent, which means that the user is not required to know all of the specific internal and external camera information or acquire additional GCPs. For the ground-to-image transformation, the defined ratios of polynomials have the forward form presented in Equations (1) and (2):

(1) $$r=\frac{\left(1\;Z\;Y\;X\cdots Y^{3}X^{3}\right)\cdot {\left(a_{0}\;a_{1}\;\cdots a_{19}\right)}^{T}}{\left(1\;Z\;Y\;X\cdots Y^{3}X^{3}\right)\cdot {\left(1\;b_{1}\;\cdots b_{19}\right)}^{T}}$$

(2) $$c=\frac{\left(1\;Z\;Y\;X\cdots Y^{3}X^{3}\right)\cdot {\left(c_{0}\;c_{1}\;\cdots c_{19}\right)}^{T}}{\left(1\;Z\;Y\;X\cdots Y^{3}X^{3}\right)\cdot {\left(1\;d_{1}\;\cdots d_{19}\right)}^{T}}$$

where r and c are image space coordinates, X,Y,Z are ground coordinates, and a, b, c, and d are the respective RFCs [46] provided by the remote sensing product vendor.

**Figure 1:**
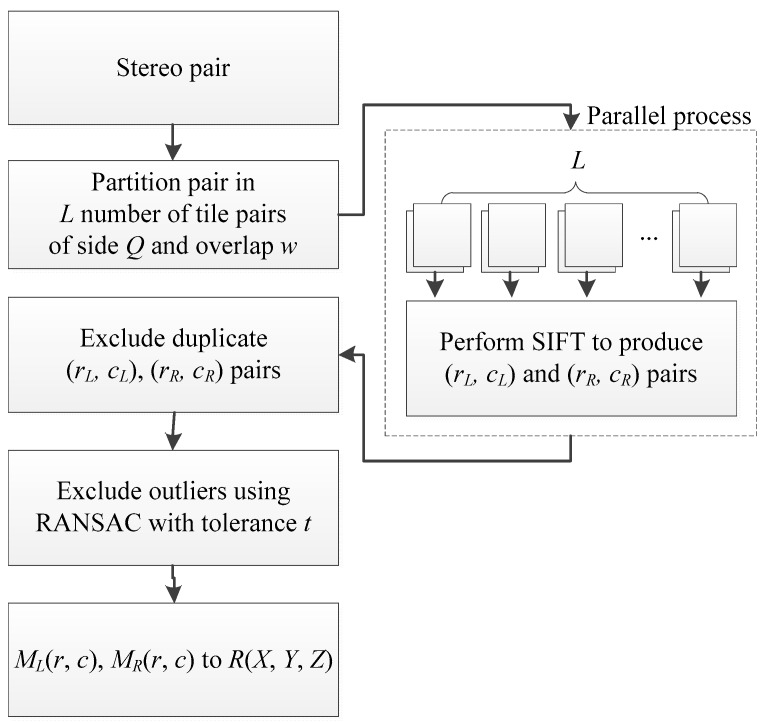
Satellite stereo-pair processing for the extraction of an elevation point cloud.

The methodology described in this paper proposes the use of SIFT in consecutive tiles of the stereo pair that are processed in parallel so as to reduce computational cost. Feature pairs $M_{R}\left(r_{R},c_{R}\right)$, $M_{L}\left(r_{L},c_{L}\right)$ are stored for each tile and controlled for duplicates at the end of the process. Then, outlier detection is performed using RANSAC. At the final steps $M_{R}\left(r_{R},c_{R}\right)$, $M_{L}\left(r_{L},c_{L}\right)$, pairs from the image domain are transformed to object space R(X, Y, Z). The methodology is outlined in Figure 1 and described in detail in the following paragraphs.

### 2.2. Cascading

**Figure 2:**
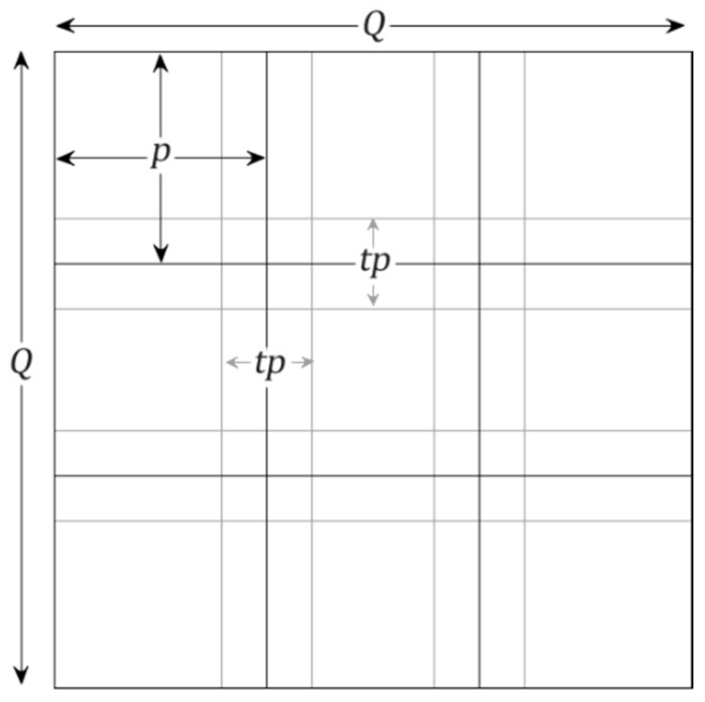
Example of cascading of a rectangular image of size Q×Q pixels broken down in tiles of size p×p that overlap by t×p.

It has long been known that program restructuring can dramatically change computational cost [47,48,49,50]. The partitioning of loop iteration space leads to the segmentation of a large matrix into smaller blocks, thus fitting accessed matrix elements into a smaller and reusable buffer. Loop tiling aims at improving cache performance, making effective use of parallel processing capabilities, and reducing the overheads associated with executing loops. The need for overlapping or cascading rather than using consecutive tiles arises from the use of a Gaussian kernel in the SIFT feature detection method that only detects features in the center of the kernel. For simplicity, the image and tiles are considered to be rectangular. For an image of size Q that can be broken down to ${\left(Q/p\right)}^{2}$ tiles of side p, an iterative computation of $Q^{2}$ pixels will take place regardless of whether the image is processed as a whole or in parts. When tiles overlap by {t×p; 0<t<1} pixels on each side, then, taking into account that the tiles in the perimeter are only overlapped once (Figure 2), the total number of iterations T is given by:

(3) $$T={\left(\frac{Q-2\left(p-\frac{t}{2}p\right)}{p-tp}+2\right)}^{2}={\left(\frac{Q-tp}{p-tp}\right)}^{2}$$

Obviously, for p<<Q and w≈1, T can cause great computational cost, and the use of parallel computing is strongly recommended.

### 2.3. SIFT Feature Point Detection and Matching

The Scale Invariant Feature Transform or SIFT [22,28] is an image descriptor for image-based matching that is computed from the image intensities around key locations obtained from the scale-space extrema of differences-of-Gaussians (DoG) [51,52]. The method of feature detection is roughly equivalent to the Laplacian of Gaussian (LoG) scale-adaptive blob detection method [53,54] that is used in a variety of environmental detection applications [55,56], as the DoG is given by the subtraction of two LoGs at different scales:

(4) $$D\left(r,c;\sigma \right)=L\left(r,c;k_{i}\sigma \right)-L\left(r,c;k_{j}\sigma \right)$$

where L(r,c;kσ) is computed from the input image M(r,c) by convolution with a Gaussian kernel of scale kσ:

(5) $$G\left(r,c;k\sigma \right)=\frac{1}{2\pi {\left(k\sigma \right)}^{2}}e^{-\left(r^{2}+c^{2}\right)/2{\left(k\sigma \right)}^{2}}$$

The characteristic of the SIFT descriptor is that it remains invariant to noise, translations, rotations, and scaling transformations in the image domain, and is robust to reasonable viewpoint and illumination variations [57]. Once SIFT descriptors are detected on the members of the stereo pair, they can be matched [22] as homologous for further processing.

### 2.4. Outlier Detection

**Figure 3:**
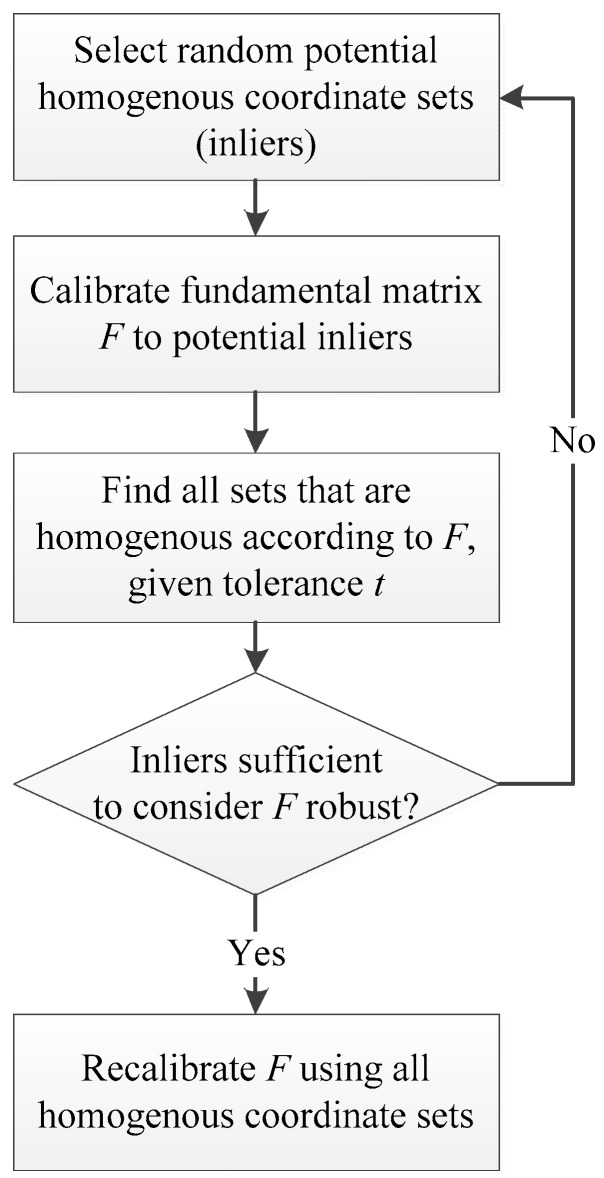
RANdom SAmple Consensus (RANSAC) algorithm for estimating homologous coordinate sets of corresponding points in a stereo image pair.

Although satellite stereo pairs are usually produced using a linear pushbroom camera [58] and therefore are not perspective images, strictly speaking, the tentative feature matches in them generate a matrix that is analogous to the fundamental matrix of perspective cameras [59] that can be explained by some set of model parameters, while false matches (outliers) do not fit that model [60]. Outliers can come, e.g., from extreme values of the noise in the images, erroneous measurements, incorrect hypotheses about the interpretation of data, shadows, etc. The RANdom SAmple Consensus (RANSAC) algorithm [61] is an iterative model parameter estimator that, unlike conventional parameter estimation techniques, uses the smallest possible set of potential inliers and proceeds to enlarge it with consistent data points, instead of pruning outliers from an initial solution. The method assumes that given a (usually small) set of inliers, a procedure to estimate the parameters of a model that optimally explains or fits this data can be attained. RANSAC is very popular to the computer vision community, as it can estimate model parameters with a high degree of accuracy, even when a significant number of outliers are present in the dataset. Figure 3 shows the steps of the RANSAC algorithm for estimating homologous coordinate sets of corresponding points in a stereo image pair. The procedure is repeated for a fixed number of iterations, each time producing an error measure that dictates whether this model is refined or needs to be rejected because of very few points being classified as inliers.

In order to robustly select homologous image coordinates $M_{i}$ and $M_{i}^{\prime }$ in a stereo image pair, the input to the RANSAC algorithm are (a) all potentially homologous pairs, (b) the parameterized model of the fundamental matrix F, which can be fitted to the coordinates pairs, and (c) the empirically chosen maximum Sampson distance t of a fundamental matrix fits with respect to a set of matched points [62]. The fundamental matrix F is a 3 × 3 matrix that relates corresponding points to stereo images so that for any pair of corresponding points $M_{i}$ and $M_{i}^{\prime }$ in both images, the epipolar constraint $M_{i}^{\prime T}FM_{i}=0$ must hold [62]. The fundamental matrix F can be computed from correspondences between image points alone, without knowledge of camera internal parameters or the relative orientation required. A relatively strict RANSAC tolerance t can remove all of the wrong matches, but also many correct matches; on the contrary, a relatively loose RANSAC threshold may keep more matches, but will not eliminate all the wrong ones [63].

### 2.5. Image to Ground

The inverse process of the ground-to-image transformation (Equations (1) and (2)) allows the user to perform photogrammetric tasks such as orthorectification and stereo reconstruction and requires mutual information matching between the stereo-pair members. Essentially, a pixel pair $\left\{M_{i},\;M_{i}^{\prime }\right\}$ representing the same object needs to be identified in order to solve Equations (1) and (2) iteratively toward the object’s real world coordinates [64,65]. Since the distance between pixels $M_{i}$ and $M_{i}^{\prime }$, which is also called disparity, is independent of pixel intensity, one band from each member of the stereo pair is usually adequate for DEM extraction.

Along with RFCs, the normalization parameters for the forward form of the RFM are provided so that the un-normalized coordinates of an object located at R(X,Y,Z) are $X=X_{n}X_{s}+X_{o}$, $Y=Y_{n}Y_{s}+Y_{o}$, and $Z=Z_{n}Z_{s}+Z_{o}$, where $X_{n}$, $Y_{n}$, $Z_{n}$ are normalized coordinates, $X_{s}$, $Y_{s}$, $Z_{s}$ are scale parameters, and $X_{o}$, $Y_{o}$, $Z_{o}$ are offset parameters. Respectively, in image space, normalization parameters exist so that an un-normalized pixel M(r, c) is equal to $r=r_{n}r_{s}+r_{o}$ and $c=c_{n}c_{s}+c_{o}$, where $r_{n}$, $c_{n}$ are normalized coordinates, $r_{s}$, $c_{s}$ are scale parameters, and $r_{o}$, $c_{o}$ are offset parameters. For both R(X,Y,Z) and M(r, c), normalization parameters are different for each member of the stereo pair. The effect of this normalization is the minimization of errors (e.g., due to decimal truncation) during computations [65]. Furthermore, use of the RFCs and normalization parameters serves for georeferencing any part of the image. The reconstruction process begins with the rough estimation of the ground coordinates [65] fed in iterative minimization of the error vector v usually with the least-squares method [66]:

(6) $$\left[\begin{array}{l} v_{rL}\\ v_{cL}\\ v_{rR}\\ v_{cL} \end{array}\right]=\left[\begin{array}{ccc} \begin{array}{l} \partial r_{L}/\partial \;Z_{n}/Z_{sL}\\ \partial c_{L}/\partial \;Z_{n}/Z_{sL} \end{array} & \begin{array}{l} \partial r_{L}/\partial \;Y_{n}/Y_{sL}\\ \partial c_{L}/\partial \;Y_{n}/Y_{sL} \end{array} & \begin{array}{l} \partial r_{L}/\partial \;X_{n}/X_{sL}\\ \partial c_{L}/\partial \;X_{n}/X_{sL} \end{array}\\ \partial r_{R}/\partial \;Z_{n}/Z_{sL} & \partial r_{R}/\partial \;Y_{n}/Y_{sL} & \partial r_{R}/\partial \;X_{n}/X_{sL}\\ \partial c_{R}/\partial \;Z_{n}/Z_{sL} & \partial c_{R}/\partial \;Y_{n}/Y_{sL} & \partial c_{L}/\partial \;X_{n}/X_{sL} \end{array}\right]\left[\begin{array}{l} \Delta Z\\ \Delta Y\\ \Delta X \end{array}\right]-\left[\begin{array}{l} r_{nL}-{\hat{r}}_{nL}\\ c_{nL}-{\hat{c}}_{nL}\\ r_{nR}-{\hat{r}}_{nR}\\ c_{nL}-{\hat{c}}_{nL} \end{array}\right]$$

where ${\hat{r}}_{L}$ and ${\hat{r}}_{R}$ are the estimated image space coordinates in each iteration. While this computation is straightforward and allows for fast convergence [67], an automatic pixel-wise matching of large stereo-pair products becomes challenging, as it requires global and local matching methods [68] to ensure robustness [16]. Global methods typically solve a single optimization problem and are extremely time-consuming for large datasets; hence, local algorithms are employed to solve per-pixel optimization, and then the entire dataset is scanned for an optimal disparity value at each pixel [69].

### 2.6. Evaluation Criteria

This above methodology generates a point cloud that can later be interpolated into a regular grid DEM. The simplest method to evaluate the point detection method is to count the number of objects R detected within a given image M, or otherwise the fraction of pixels covered by the detection algorithm. Spacing among the resulting points R is accessed for irregularity by triangulating, so that no point in R is inside the circumcircle of any of the derived triangles T(R), which is a process known as Delaunay triangulation. After eliminating the duplicate sides of T(R), the average length, or spacing irregularity, of the remaining sides $T^{\prime }\left(R\right)$ is estimated as well as the first, second and third quartiles of lengths (i.e., the middle value between the minimum and the median of the dataset, the median, and the middle value between the median and the maximum of the dataset). It can be shown that the minimum average spacing irregularity among points occupying nodes of a regular grid converges to (but never reaches) the resolution of the grid times $\sqrt{2}$ as the number of points increases. Finally, the quality of each DEM product against a reference DEM can be estimated using the average error $\bar{e}=\sum \left(\hat{z_{i}}-z_{i}\right)/N$, the average relative error $\bar{e_{r}}=\sum \left(\hat{z_{i}}-z_{i}\right)/Nz_{i}$, the Root Mean Square Error (RMSE) defined by $\sqrt{\sum {\left(\hat{z_{i}}-z_{i}\right)}^{2}/N}$ where $z_{i}$ is the elevation of the measured point i considered as ground truth, $\hat{z_{i}}$ is the elevation of point i on each DEM, and N is the number of measurements.

## 3. Case Study

**Figure 4:**
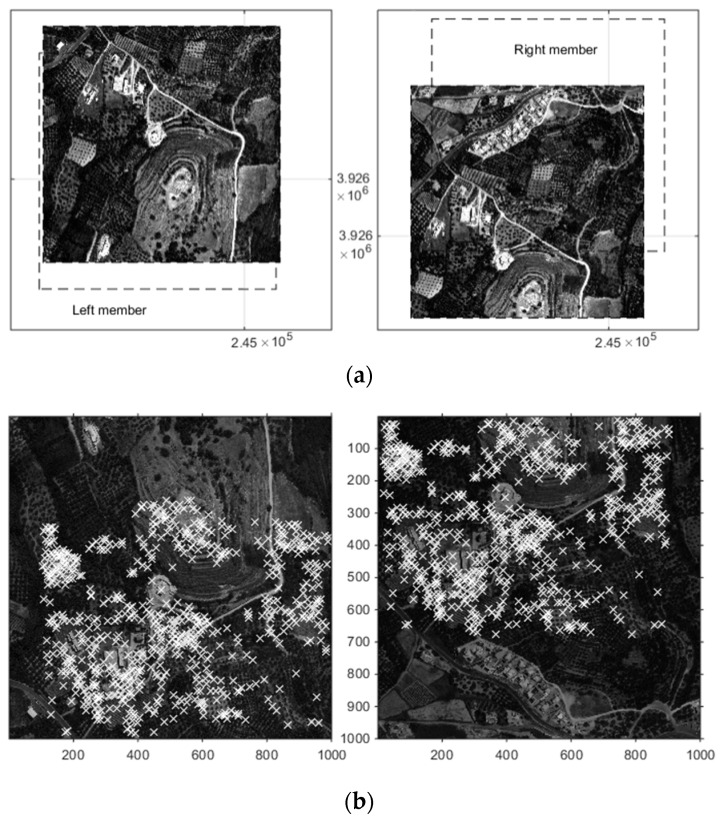
Members of a sample Geoeye-1 stereo pair from Almirida watershed. Images are shown at original positioning, and dashed lines show their respective location after rough georeferencing with their Rational Function Models (RFMs) (**top pair**). A fast image matching is used to determine homologous features (**bottom pair**). Axes units in pair (**a**) in m of the Greek National Grid coordinate system. Axes units in pair (**b**) in pixels.

The Geoeye-1 GeoStereoTM stereo pair used in this study was acquired on 13 August 2009 over the wider area of Almirida watershed in Crete, Greece [16]. The product is characterized as Panchromatic—Multispectral, has an 0.5-m pixel size, and the two members were collected at nominal azimuths of 9.1159° and 194.5472° degrees, respectively, and nominal elevations of 79.55334° and 62.05786°, respectively [9,16]. A sample of members from a small area of Almirida watershed stereo-pair images is shown in Figure 4, which were roughly georeferenced using their respective RFMs. The sample is a 1000 px × 1000 px image block that translates to a 500 m × 500 m area of rough, hilly terrain. The area is sparsely vegetated with olive trees, natural shrubs, and a few conifers. The dominant feature of the sample area is a calcic hill scarred by a series of now abandoned planting terraces.

In order to compare DEMs to ground truth, 360 control points were measured using a Total Station and georeferenced using a differential Global Positioning System (GPS) network. For reference, a 2-m resolution DEM produced using v10.1 of ERDAS^®^ IMAGINE software (Leica Geosystems, Atlanta, GA, USA) using the same Geoeye-1 stereo pair and a 5-m commercial DEM produced from aerial photography stereo pairs were also compared with the ground truth measurements. Prior to comparison, a single GCP within the study site is used to compensate for shift terms and achieve accurate absolute geopositioning (Fraser and Ravanbakhsh, 2009; Fraser and Yamakawa, 2003).

## 4. Results and Discussion

**Figure 5:**
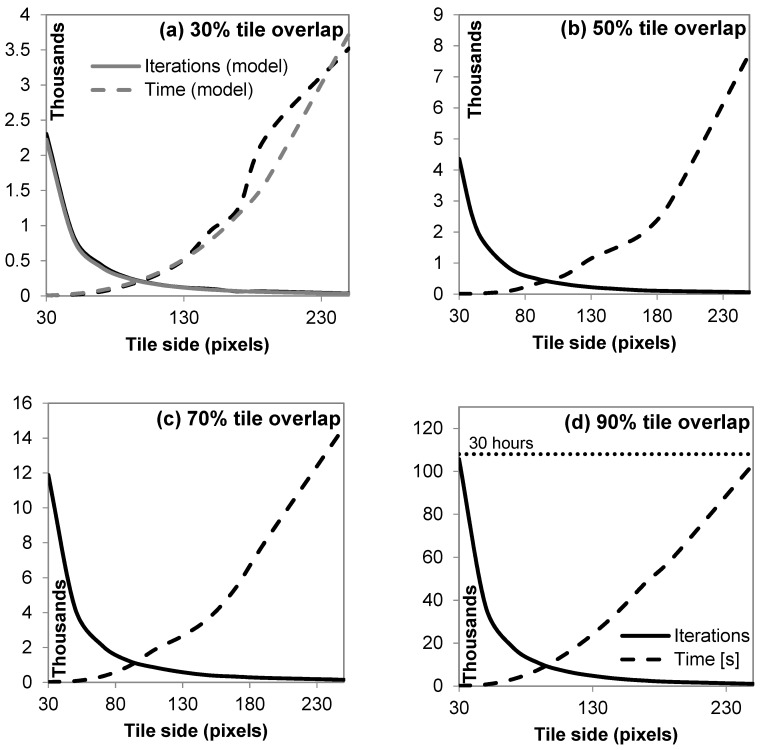
Resulting iterations and computational processing unit (CPU) time [s] from the cascading of rectangular tiles with side p ranging from 30 to 250 pixels and overlap of 30 to 90%.

As expected from Equation (6), the number of iterations needed to process all the tiles decreases with the tile side (Figure 5). The decrease in every case can be modeled well with a negative power equation of the form $y=ax^{-2}$, where *y* is the number of iterations, and *x* is the number of pixels. Figure 5a shows such an example model for $a=2\times 10^{6}$ fitting data with $R^{2}=0.96$. For a specified tile overlap fraction, the processing time increases at a rate that can be approximated with a power equation of the form $y=ax^{3}$, where *y* is time in seconds and x is the number of pixels. Figure 5a shows an example model for $a=2.38\times 10^{-4}$ fitting data with $R^{2}=1.00$. Intuitively, one would expect that since the tiles are square, and each pixel requires a set time to process, the processing time would merely be proportional to the square of the number of pixels. Nevertheless, additional overhead related to memory use and side processes increases this estimate to the number of pixels cubed. For the processing power used in this study (a PC equipped with an Intel i7@2.67 GHz multithreaded to run up to seven processes in parallel), time starts becoming an issue for large tiles and large overlaps, when essentially the larger portion of information is processed linearly rather that in parallel. Thus, starting from a few seconds to process the image broken down to 30 px × 30 px tiles, the combination of 250 px × 250 px tiles at 90% overlap can occupy the processor for almost 30 h (Figure 5d).

**Figure 6:**
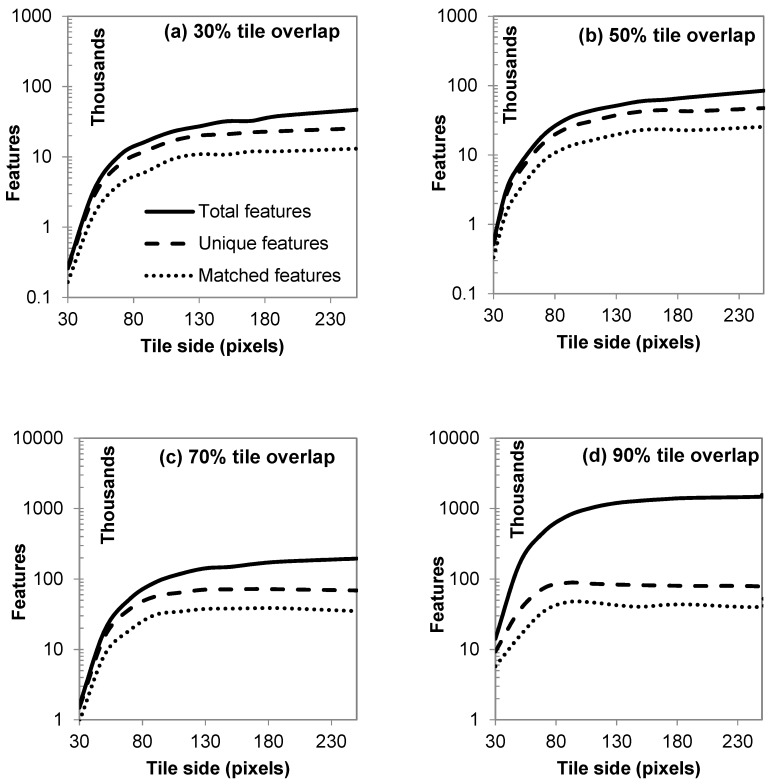
Number of resulting total, unique, and tentative feature matches (logarithmic scale) from cascading of rectangular tiles with side p ranging from 30 to 250 pixels and an overlap of 30% to 90%.

Detected features increase logarithmically (Figure 6) as the tile size increases, following an asymptotic equation of the form y=ln(x)−b. Therefore, a practical limit can be set for the maximum sensible tile size that will produce enough detected features without abusing computational processing unit (CPU) time (Figure 5). From Figure 6, it can be inferred that for a specified cascade, a tile size of 130 px × 130 px can be safely selected for a representative number of detected features. The percentage of total features detected for the 130-px tile side over the total features for 250 px is 58%, 61%, 73%, and 81%, respectively for tile overlaps of 30%, 50%, 70%, and 90% (Figure 6). Similarly, the increase of fraction of tile overlapping increases the number of total detected features, but over a certain threshold, new features are only duplicates. This becomes obvious in Figure 6c,d, where unique features (SIFT descriptors) are about 80,000 for both cases, while total features increase from 0.2 to 1.4 million. The additional computational cost going toward the detection of redundant features can be saved by keeping a moderate tile overlap.

**Figure 7:**
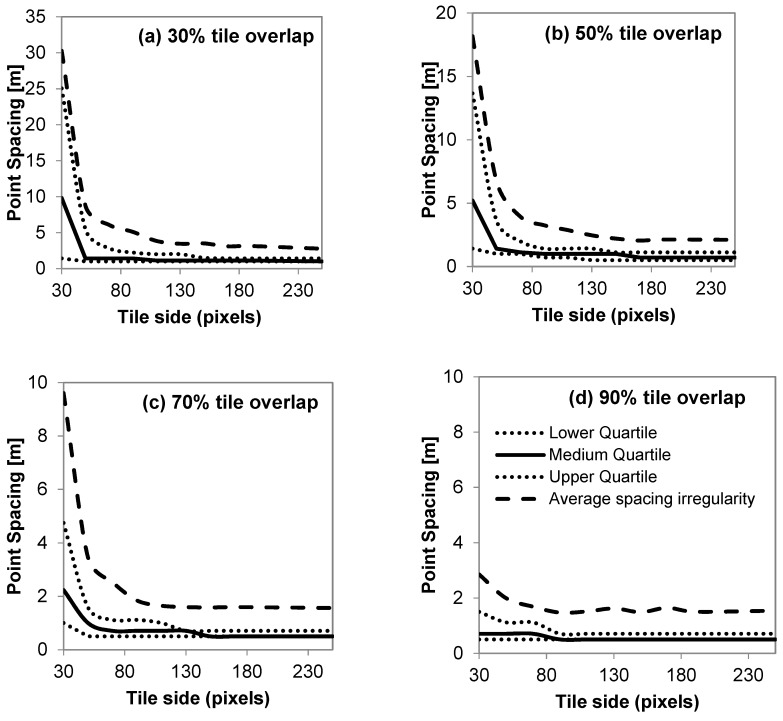
Resulting first, second, and third quartiles and average spacing irregularity of point matches from cascading of rectangular tiles with side p ranging from 30 to 250 pixels and overlap of 30% to 90%.

**Figure 8:**
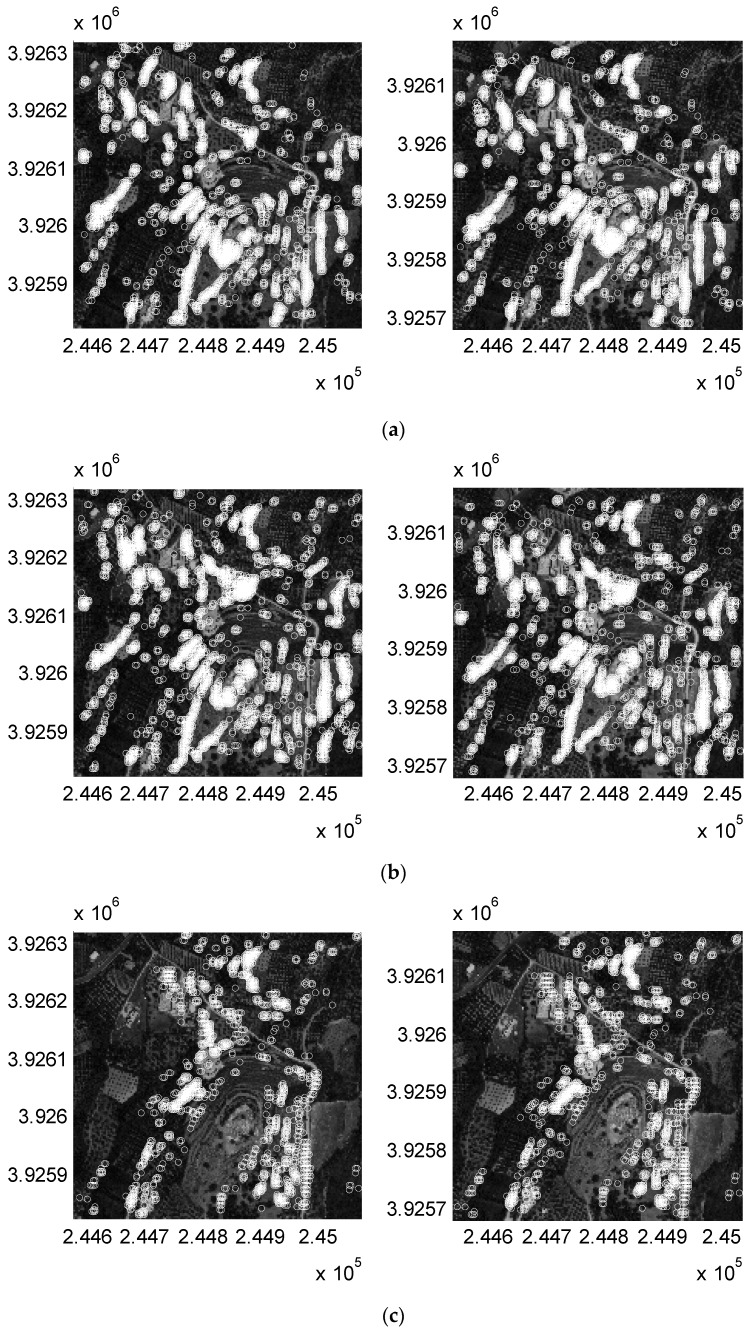
Homologous matches produced by iterative Scale-Invariant Feature Transform (SIFT) on tile sides of (**a**) 250 pixels, (**b**) 130 pixels, and (**c**) −50 pixels at 70% tile overlap and threshold t = 10^−6^ the red band of stereo pair members (one pear per row). Axes units in m of the Greek National Grid coordinate system.

Regarding spacing irregularity, it is evident that as the tile size and tile overlap increase, so do all of the spacing metrics (Figure 7). In particular, the lower and medium quartile of point spacing converge very fast to the actual resolution of the image (0.5 m) for all the tested cases. On the other hand, the upper (third) quartile of point spacing converges slower, showing that larger tiles are required in order to achieve satisfactory image coverage. The minimum possible average spacing irregularity for the resolution used in the case study can be assessed to $>0.5\times \sqrt{2}\approx 0.71$ m; therefore, any results that are close to this value can be considered optimal. For the selected sample, the minimum average spacing irregularity is equal to 1.47 m, and was achieved for a tile side of 90 px and 90% tile overlap. Nevertheless, an average spacing of under 1.6 m can also be achieved for tile sizes of 130 px × 130 px at a 70% overlap. Therefore, the additional CPU cost that is required leads to no profit. Considering that the average spacing irregularity can be related to resolution, a value of 1.6 m approximates a final resolution of $1.6/\sqrt{2}=1.13$ m, which is rather adequate for a wide range of applications. Figure 8 shows the resulting homologous points estimated by the method for 70% tile overlap and three different tile sizes, with a 130-px tile size and 70% overlap producing the optimal results regarding point spacing and CPU load. At lower tile sizes (Figure 8c), matched points become irregularly spaced, cluttering around high contrast objects, while larger (Figure 8a) tiles yield no significant number of additional points. It is also worth mentioning that enabling parallel computation (in this case using a pool of seven CPU threads) cuts down processing time from 2 h to 45 m using the same hardware.

**Figure 9:**
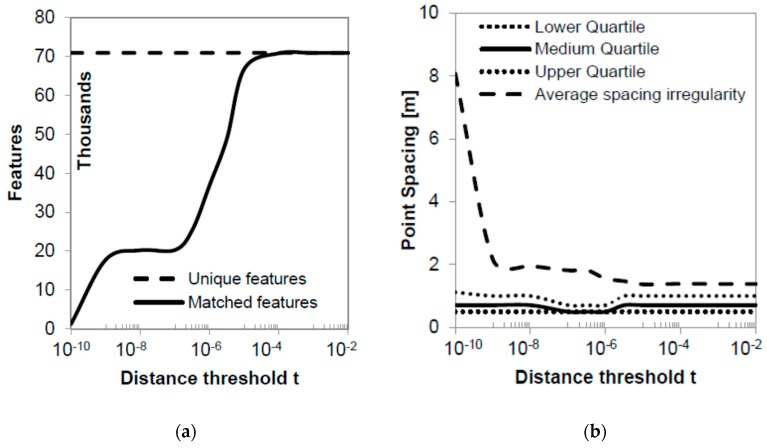
(**a**) Number of resulting unique and tentative feature matches and (**b**) homologous feature spacing statistics for different values of RANSAC distance threshold t on a logarithmic scale.

**Figure 10:**
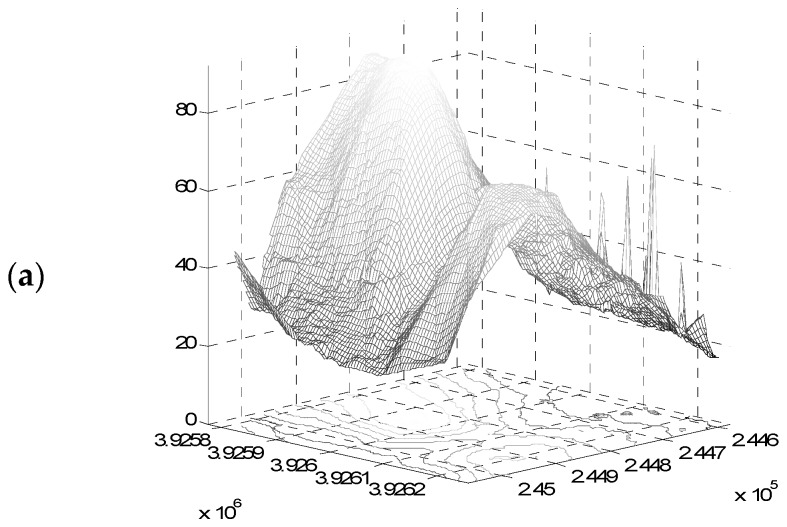

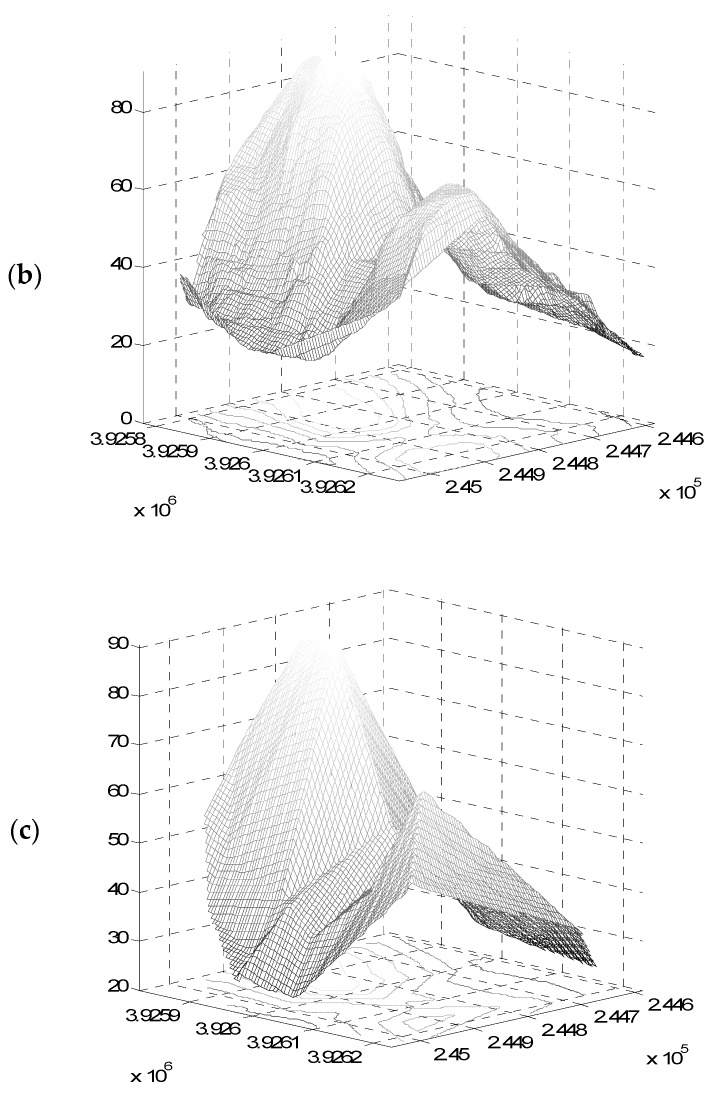
DEMs created for RANSAC distance thresholds t equal to (**a**) 10^−2^, (**b**) 10^−6^, and (**c**) 10^−10^.

Optimization for the RANSAC distance threshold t results are shown in Figure 9. In order to successfully calibrate this parameter, resulting point clouds are interpolated to 1.5-m resolution DEMs and checked for obvious errors. Figure 10 shows selected DEMs that were resampled to 5 m for better clarity. For values over 10^−4^, the RANSAC method has little or no effect on the filtering of feature points, assuming all of them are homologous. Nevertheless, the results display several outliers that cause irregular spike-like artefacts in the DEM (Figure 10a). For values of distance threshold between 10^−5^ and 10^−7^, the number of candidate homologous feature points drops fast down to 30% of the unique features originally detected in the stereo pair. As t decreases more, points become irregularly spaced (Figure 10b), and the produced DEM (Figure 10c) becomes less detailed, showing steep surfaces as a result of the linear interpolation used to produce it. At these values of threshold t, RANSAC acts as an overtuned high-pass filter eliminating true information. Therefore, a good approximation for the optimum t threshold is 10^‒6^, which produces an acceptable average spacing irregularity of 1.55 m (Figure 9b) and a smooth DEM with a high level of detail and no apparent outliers (Figure 10b). For this value, the homologous detected pixels are 37,000, representing 3.7% of the 1-M pixel sample.

**Figure 11:**
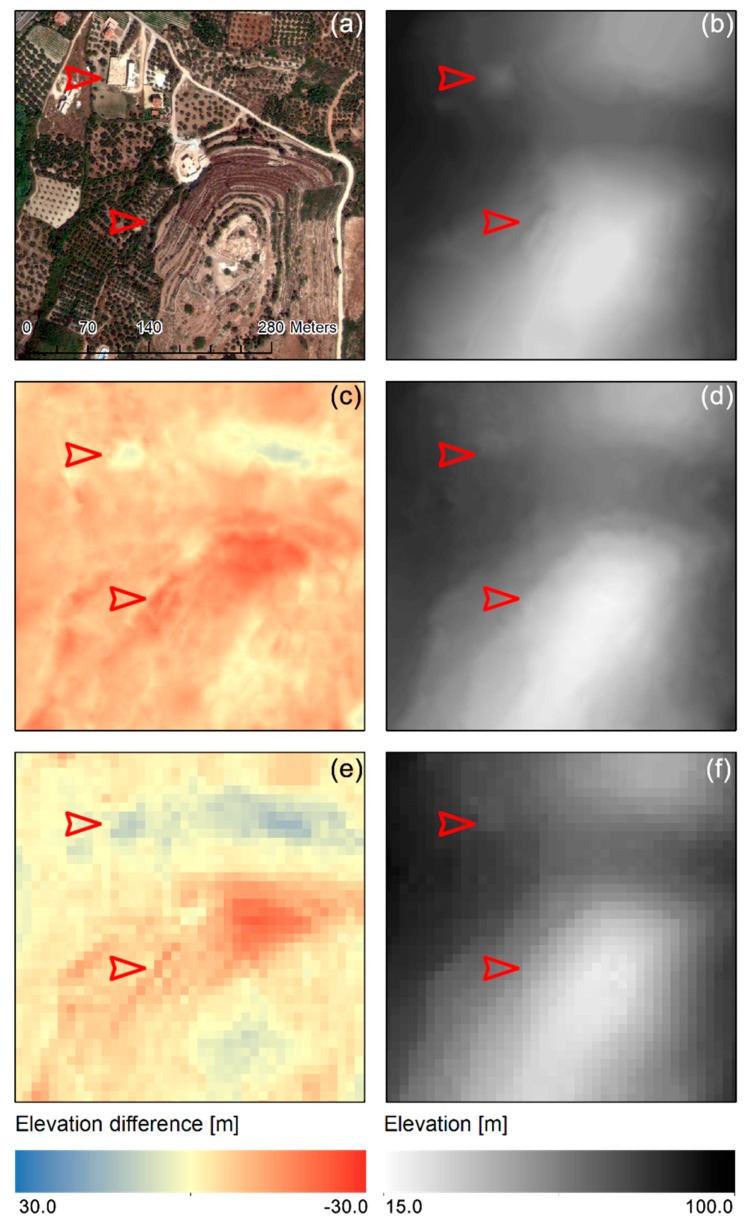
(**a**) Part of the Geoeye-1 sample used in the study and (**b**) visual comparison of produced 1.5-m Digital Elevation Models (DEM) with (**d**) reference 2-m DEM produced from the Geoeye-1 using ERDAS^®^ and (**f**) 5-m commercial DEM from aerial photo stereo pair, and difference between the produced 1.5-m DEM and the reference 2-m DEM (**c**), as well as the 5-m commercial DEM (**e**). Red arrows denote areas of interest for comparison.

Finally, the elevation values in the point cloud are transformed to a 1.5-m DEM using linear interpolation. The resulting DEM for the sample area used in this case study (Figure 11a) is shown in Figure 11b. The 1.5-m sample DEM values range from 22.56 m to 90.36 m with a mean elevation of 53.26 m (Table 1). Visually compared to the 2-m DEM (Figure 11d) and the 5-m DEM (Figure 11f), the new DEM shows superior detail, depicting building shapes and landforms with higher contrast (red arrows in Figure 11). The 2-m DEM also lays on average −5.42 m lower than the new DEM (Table 1), with differences ranging from −14.50 to 4.89 m (Figure 11c). The 5-m DEM lays on average −1.57 m lower than the new DEM (Table 1), with elevation differences ranging from −14.05 to 9.03 m (Figure 11e). Overall, with respect to the 1.5-m DEM, the 2-m DEM underestimates the elevation of most of the sample except valleys, while the 5-m DEM underestimate ridges and slightly overestimates valley elevation (Figure 11c,e). Assessment of the DEM quality is achieved using the goodness of fit criteria shown in Table 1. For the study area, the coefficient of determination R2 for all cases was above 0.99, meaning that in terms of goodness of fit, the quality of DEMs are generally good. A reduction of about 70% is observed in the $\bar{e}$ and $\bar{e_{r}}$ when moving from the 5-m DEM to the 2-m and 1.5-m DEMs. Furthermore, for the sampled area, the RMSE of both 2-m and 1.5-m DEMs compared with Total Station measurements is close to 1 m, with the new DEM being 2 cm more accurate, while the 5-m commercial DEM yields an RMSE of roughly 2 m.

## 5. Conclusions

A module for DEM extraction from satellite stereo pairs was developed in MATLAB (MathWorks Inc., Natick, MA, USA) and applied in Crete using a Geoeye-1 0.5-m product. The module uses a combination of SIFT and RANSAC run in parallel computing mode to perform the detection of tentative feature matches in the stereo pair. A simple method to successfully calibrate the RANSAC algorithm in order to achieve optimal results is also shown. The DEM resolution that can be achieved using a specific point cloud is determined using the average spacing irregularity in the point cloud. Besides work by the same authors [40,41,42], to our knowledge, the combination of methods used has not been documented in the literature for Geoeye-1 applications.

During the module’s optimization, it is shown that today’s parallel processing enabled software and hardware that significantly decrease the processing cost of data and CPU-intensive processes such as DEM extraction. By segmenting the original image into smaller tiles, the developed module makes use of this advantage and reduces processing time for SIFT feature detection to a fraction of the original (Figure 5). This has a positive impact in the achieved DEM quality versus the CPU time spent.

Using these methods, the modules detect the required number of matching points to achieve 1.5-m resolution for subsequent DEM extraction. The 1.5-m DEM that is produced is superior in terms of depicted land surface details, as well as the calculated metrics when compared against a 2-m DEM produced using ERDAS^®^, which served as a benchmark for this study (Table 1). The results from the statistical analysis (RMSE and error values) undertaken to investigate the accuracy of the 1.5-m DEM by comparing the Total Station elevations at 360 points with on-ground field survey elevations indicate that the 1.5-m satellite stereo pair DEM adequately represents the ground elevations for any detailed environmental modeling application.

The module can be used with other stereo-pair products accompanied by the respective RFM information. The module is designed to allow automated matching point detection with minimal parameterization and can thus be operated by non-experts for the production of Very High Resolution (VHR) DEMs for environmental applications. Future versions will deal with current limitations such as mismatched points due to repeated structures [70] that tend to cause algorithms for epipolar geometry estimation to fail.

## Figures and Tables

**Table 1:** Statistics and goodness of fit metrics of the two reference DEMs (5-m and 2-m resolution) and the newly produced DEM (1.5-m resolution).

	5-m DEM	2-m DEM	1.5-m DEM
**Min value [m]**	19.02	26.72	22.56
**Max value [m]**	93.95	96.86	90.36
**Mean value [m]**	53.00	58.69	53.26
**St. dev. [m]**	19.04	17.85	17.70
**Min difference from 1.5 m DEM [m]**	−14.05	−14.50	-
**Max difference from 1.5 m DEM [m]**	9.03	4.89	-
**Mean difference from 1.5 DEM [m]**	−1.57	−5.42	-
**St. dev. of difference from 1.5 DEM [m]**	3.24	2.53	
$\bar{e}$ **[m] from Total Station field measurements**	−1.56	0.59	−0.45
**St. dev. of** $\bar{e}$ **[m] from Total Station**	1.18	1.02	1.00
$\bar{e_{r}}$ **[%] from the Total Station**	−2.65%	0.83%	−0.86%
**St. dev. of** $\bar{e_{r}}$ **[%] from Total Station**	2.02%	1.65%	1.64%
**RMSE from the Total Station**	1.96	1.18	1.10
**R^2^ from the Total Station**	0.9911	0.9948	0.9938
